# A Rare Consequence of Digital Isolation: Bilateral Femoral Neck Fractures Induced by Hypocalcemic Seizure in a Hikikomori Adolescent

**DOI:** 10.7759/cureus.110085

**Published:** 2026-06-01

**Authors:** Yasin Yildiz, Sevtap Barca

**Affiliations:** 1 Faculty of Medicine, Department of Pediatrics, Recep Tayyip Erdoğan University, Rize, TUR

**Keywords:** femur fracture, hikikomori, hypocalcemia, hypocalcemic seizure, vitamin d deficiency

## Abstract

Prolonged social withdrawal, conceptualized as Hikikomori, is increasingly recognized for its potential impact on physical health in adolescents because of profound lifestyle alterations. This report describes the case of a 16-year-old male patient who sustained bilateral femoral neck fractures following a hypocalcemic seizure, potentially associated with prolonged indoor confinement and lack of sunlight exposure. The patient presented to the emergency department following a first-time generalized tonic-clonic seizure. Laboratory investigations revealed profound hypocalcemia (5.3 mg/dL), severe vitamin D deficiency (5.7 ng/mL), and secondary hyperparathyroidism (219 pg/mL), leading to a diagnosis of nutritional rickets/osteomalacia. Despite initial metabolic stabilization, persistent hip pain and impaired mobility prompted further imaging. Radiographic and magnetic resonance imaging (MRI) assessments identified bilateral displaced femoral neck fractures. A detailed psychosocial history revealed that the patient had been in a state of severe social withdrawal for the preceding three years, confined to a single room with negligible sunlight exposure. A Hikikomori Questionnaire (HQ-25) score of 70 further characterized the severity of his social isolation. This case illustrates a potential clinical association between Hikikomori behavioral patterns and significant metabolic bone disturbances. Although bilateral femoral neck fractures are a rare complication of convulsive episodes, the underlying skeletal fragility in this patient may have been exacerbated by chronic, isolation-related vitamin D deficiency. This report suggests that extreme social withdrawal should be considered a potential contributing factor in atypical presentations of metabolic bone disease in the modern digital era.

## Introduction

Metabolic disturbances account for 10%-15% of pediatric generalized seizures, with hypocalcemia remaining a critical etiology [1,2]. Hypocalcemia, defined as a total serum calcium level < 8.5 mg/dL or an ionized calcium level < 1.1 mmol/L, presents with a broad clinical spectrum [2]. While hypocalcemic seizures are predominantly observed during infancy, their occurrence during adolescence is clinically atypical and often suggests profound chronic nutritional deficiency or severe environmental factors. Globally, nutritional rickets and vitamin D deficiency are the primary drivers of these metabolic crises [3]. However, in the modern digital era, extreme social withdrawal, characterized by spending virtually the entire day indoors, may be a significant contributor to severe vitamin D deficiency.

Hikikomori, a term derived from Japanese, is characterized by severe social isolation and home confinement persisting for at least six months [4]. Although initially identified in Japan, it is now recognized as a global public health concern, with reported prevalence rates of approximately 1.2% in Japan and 1.9% in Hong Kong [5,6]. Current research on Hikikomori predominantly focuses on psychosocial comorbidities, such as depression and Internet Gaming Disorder; however, the associated physical health complications remain insufficiently investigated.

The lack of UV-B exposure inherent in prolonged social withdrawal can impair cutaneous vitamin D synthesis, potentially leading to secondary hyperparathyroidism and compromised bone mineralization [7]. In this weakened skeletal state, the forceful muscular contractions associated with seizures may result in rare orthopedic injuries, such as bilateral femoral neck fractures [8]. Although post-seizure bilateral fractures have been documented in patients with malabsorption syndromes or renal osteodystrophy, their association with extreme social withdrawal has rarely been reported.

In this report, we illustrate the clinical impact of severe social withdrawal (Hikikomori) in a patient presenting with symptomatic hypocalcemic seizures and subsequent bilateral femoral neck fractures associated with critical vitamin D deficiency.

## Case presentation

A 16-year-old male patient, weighing 80 kg (body mass index (BMI): 26.1 kg/m²; BMI SDS: +1.68), with no prior history of seizures, was admitted to the emergency department following a generalized tonic-clonic seizure at home. On physical examination, the patient was post-ictal and unconscious, with a pulse rate of 132 bpm, blood pressure of 120/80 mmHg, and oxygen saturation of 92%. According to the history obtained from the family, the episode involved intense muscular contractions consistent with a generalized seizure rather than carpopedal spasms. There was no history of physical trauma or abuse. Initial laboratory investigations revealed a white blood cell (WBC) count of 11.25 × 10³/µL, hemoglobin of 13.5 g/dL, platelet count of 310 × 10³/µL, and blood glucose of 107 mg/dL. Critical biochemical findings included profound hypocalcemia, with a total serum calcium of 5.3 mg/dL (reference range: 8.5-10.5 mg/dL) and ionized calcium of 0.76 mmol/L (reference range: 1.05-1.30 mmol/L). Other serum electrolytes and biochemical markers were within normal limits. Emergency management was initiated with an immediate intravenous (IV) bolus of one ampule of 10% calcium gluconate, followed by infusion of two ampules of 10% calcium gluconate in 100 mL of 0.9% saline.

Subsequently, the patient was admitted to the pediatric ward for further evaluation. He was closely monitored and re-evaluated because of persistent hip pain and inability to move his lower extremities, and a comprehensive neurological examination was performed. The patient’s mental status was intact; he was fully conscious, oriented, and cooperative. Gross cranial nerve examination and bilateral pupillary light reflexes were normal. On motor examination, the upper extremities demonstrated normal and symmetric muscle tone and bulk, with muscle strength graded as 5/5. Although detailed motor evaluation, gait analysis, and ambulation could not be fully assessed because of severe tenderness and bilateral femoral neck fractures, no focal neurological deficits, signs of lateralization, or meningeal irritation were observed following the seizure. Deep tendon reflexes were normoactive, and plantar responses were flexor bilaterally. Coordination tests, including rapid alternating movements, were performed successfully without dysmetria or dysdiadochokinesia. Clinical signs associated with hypocalcemia, specifically Chvostek and Trousseau signs, remained negative. Follow-up laboratory tests demonstrated a markedly elevated parathyroid hormone (PTH) level of 219 pg/mL (reference range: 15-65 pg/mL), a critically low 25-hydroxyvitamin D level of 5.70 ng/mL (reference range: 20-50 ng/mL), serum calcium of 6.73 mg/dL, ionized calcium of 0.90 mmol/L, phosphorus of 5.05 mg/dL (reference range: 2.8-5.5 mg/dL), alkaline phosphatase (ALP) of 393 U/L (reference range: 44-147 U/L), and lactate dehydrogenase (LDH) of 511 U/L (reference range: 105-233 U/L). Renal function parameters were within normal limits (Table 1).

**Table 1:** Laboratory parameters and biochemical findings of the patient

Laboratory parameter	Admission level	Reference range
Glucose (mg/dL)	107	70-105
Urea (mg/dL)	22	17-43
Creatinine (mg/dL)	0.58	0.60-1.10
Aspartate aminotransferase (AST) (U/L)	26	0-35
Alanine aminotransferase (ALT) (U/L)	18	0-35
Sodium (Na) (mmol/L)	138	135-145
Potassium (K) (mmol/L)	3.8	3.5-5.1
Total calcium (Ca) (mg/dL)	5.3	8.8-10.6
Ionized calcium (mg/dL)	0.76	1.05-1.3
Albumin (g/dL)	4.2	3.5-5.2
Corrected calcium (mg/dL)	5.4	8.8-10.6
Magnesium (Mg) (mg/dL)	1.8	1.6-2.6
Phosphorus (P) (mg/dL)	5.05	2.8-5.5
Alkaline phosphatase (ALP) (U/L)	511	110-330
25-hydroxyvitamin D (25(OH)D) (ng/mL)	5.7	30-100
Parathyroid hormone (PTH) (pg/mL)	219	15-65
White blood cell (WBC) (10³/µL)	11.25	4.0-10.5
Hemoglobin (Hb) (g/dL)	13.5	13.0-16.0
Platelets (PLT) (10³/µL)	310	150-450

A diagnosis of hypocalcemic rickets/osteomalacia secondary to severe vitamin D deficiency was established in consultation with pediatric endocrinology. Treatment was initiated with oral calcium carbonate (2,500 mg) and cholecalciferol (880 IU vitamin D3) administered three times daily, supplemented with vitamin D3 drops (2,400 IU/day). Despite metabolic stabilization, the patient exhibited persistent anhedonia, dysphoria, and an inability to stand or ambulate. Physical examination revealed marked tenderness over both hips. Plain radiography confirmed bilateral femoral neck fractures (Figure 1). Magnetic resonance imaging (MRI) of the hips further demonstrated bilateral displaced femoral neck fractures on T1-weighted coronal sequences (Figures 2a, 2b). Because emergent orthopedic intervention was required and timely familial consent could not be obtained, bone densitometry (DEXA) was not performed preoperatively.

**Figure 1:**
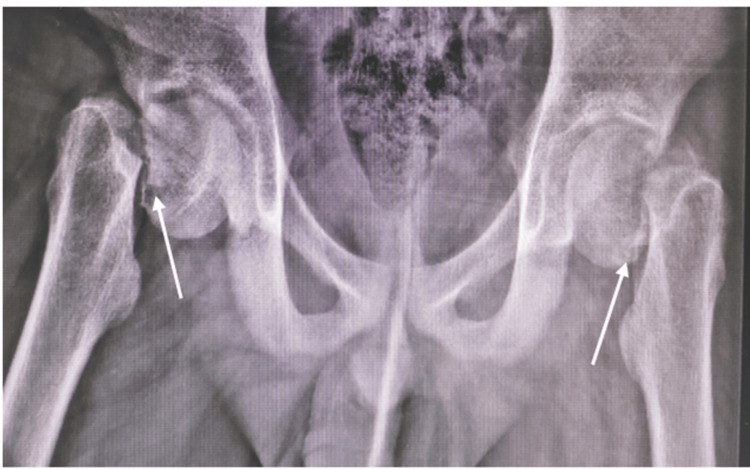
Bilateral femoral neck fractures (white arrow)

**Figure 2:**
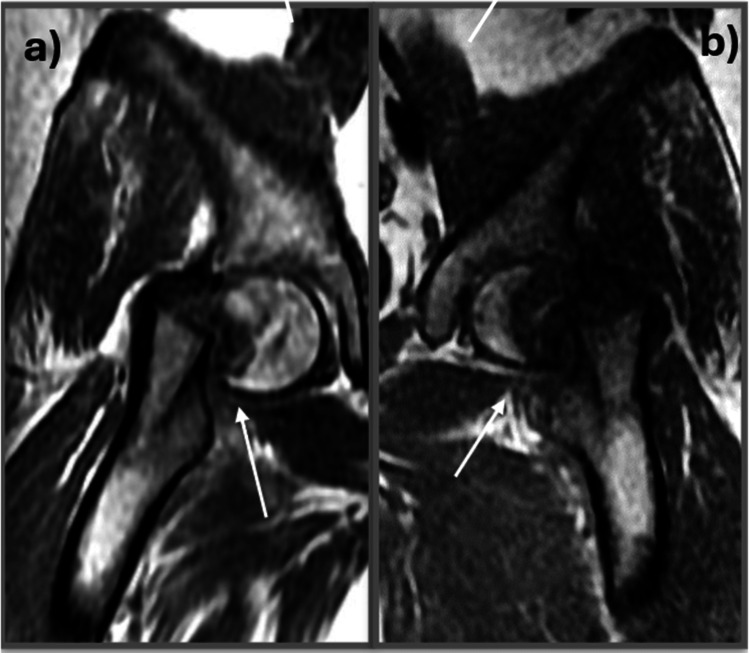
T1-weighted coronal MRI images of the right (a) and left (b) hip joints, demonstrating bilateral displaced femoral neck fractures

A detailed psychosocial history obtained postoperatively revealed that the patient was severely introverted and had remained in a state of near-total home confinement for more than three years. He had withdrawn from all social interactions outside his immediate family, with whom communication had also become markedly limited. His daily routine was characterized by prolonged isolation in his room with the curtains drawn, excessive screen time, and a sedentary lifestyle. His diet consisted predominantly of fast food, contributing to significant weight gain. The patient attributed the onset of his withdrawal to the COVID-19 pandemic, which subsequently evolved into a chronic pattern of social isolation. Although the family did not experience direct bereavement during the pandemic, they reported a significant decline in the patient’s mood and social engagement following the initial lockdowns. He had no prior psychiatric evaluation or history of psychotropic medication use. Assessment using the 25-item Hikikomori Questionnaire (HQ-25) [9] yielded a score of 70, supporting a diagnosis of severe pathological social withdrawal consistent with Hikikomori. Written informed consent was obtained from the patient’s legal guardians for publication of this case report and the accompanying images.

## Discussion

The primary clinical significance of this case report lies in the potential association between Hikikomori, a modern phenomenon of severe social withdrawal, and significant physical injury. Although bilateral femoral neck fractures secondary to generalized seizures have been documented, this case highlights how extreme digital isolation may contribute to severe metabolic bone disease in young individuals.

A hallmark of disorders related to technology addiction is prolonged screen exposure. Excessive sedentary behavior and lack of physical activity in indoor environments can lead to multisystem complications, including musculoskeletal and nutritional disturbances [10]. Our patient’s three-year period of isolation, supported by an HQ-25 score of 70, underscores how such behavioral patterns can evolve from a social condition into a clinically significant pathological state.

Previous literature suggests that increased screen time in adolescents may be associated with lower serum vitamin D concentrations, potentially because of reduced outdoor activity and limited sunlight exposure [7,11]. Conversely, higher serum vitamin D levels have been observed in physically active adolescents with limited screen time [12]. In the present case, vitamin D deficiency likely developed because of impaired cutaneous synthesis related to prolonged indoor isolation and minimal sunlight exposure. This deficiency may have led to reduced intestinal calcium absorption and subsequent secondary hyperparathyroidism. Elevated PTH levels likely promoted bone resorption to maintain calcium homeostasis, contributing to impaired osteoid matrix mineralization, a process consistent with adolescent osteomalacia. Furthermore, the patient’s sedentary lifestyle and calcium-poor diet, characterized by frequent fast-food consumption, may have acted as additional contributing factors by reducing mechanical weight-bearing activity necessary for normal bone health [13].

Simultaneous bilateral femoral neck fractures are rare in young individuals and are typically associated with high-energy trauma or underlying metabolic disorders such as renal osteodystrophy, malabsorption syndromes, or endocrine disease [14,15]. The markedly elevated ALP level and profound hypocalcemia observed in our patient suggest severely impaired bone mineralization, predisposing the skeletal system to fractures during hypocalcemic convulsions [8,16].

Differential diagnosis excluded other etiologies of hypocalcemia [3]. Electrolyte disturbances such as hypomagnesemia and renal failure were ruled out through biochemical assessment. Elevated PTH levels supported secondary hyperparathyroidism and effectively excluded primary hypoparathyroidism. Furthermore, the absence of gastrointestinal symptoms and normal liver and renal function tests suggested that the vitamin D deficiency was predominantly environmental rather than secondary to organic disease. The relatively elevated serum phosphorus level observed at admission was interpreted in the context of age-specific reference ranges and the possible release of intracellular phosphate following generalized seizure activity.

Nevertheless, this study has several limitations. First, the emergent nature of the bilateral femoral neck fractures required immediate surgical intervention, precluding objective assessment of bone mineral density using DEXA during the preoperative period. Second, advanced serological testing (e.g., anti-gliadin or anti-endomysial antibodies) to definitively exclude gastrointestinal malabsorption, as well as molecular analyses for genetic bone disorders, were not prioritized because the clinical presentation was strongly consistent with severe environmental deprivation related to Hikikomori, in addition to limitations related to familial consent. Finally, further evaluation for renal calcium loss, such as urinary calcium-to-creatinine ratio measurement, was considered to have limited diagnostic utility after the initiation of intensive hydration and calcium replacement during acute management.

## Conclusions

In conclusion, this case provides insight into how prolonged digital isolation may be associated with systemic metabolic disturbances leading to surgical emergencies. Clinicians should consider social lifestyle factors and sunlight exposure in adolescents presenting with seizures accompanied by hypocalcemia or unexplained musculoskeletal complaints. Consequently, Hikikomori-like social withdrawal may represent a potential contributing factor in the clinical evaluation of metabolic bone disease in modern practice.

## References

[1] Castilla-Guerra L, del Carmen Fernández-Moreno M, López-Chozas JM, Fernández-Bolaños R (2006). Electrolytes disturbances and seizures. Epilepsia.

[2] Wilfong A (2026). Seizures and epilepsy in children: classification, etiology, and clinical features. UpToDate.

[3] Bharill S, Wu M (2023). Hypocalcemia and hypercalcemia in children. Pediatr Rev.

[4] Wong JC, Wan MJ, Kroneman L, Kato TA, Lo TW, Wong PW, Chan GH (2019). Hikikomori phenomenon in East Asia: regional perspectives, challenges, and opportunities for social health agencies. Front Psychiatry.

[5] Kato TA, Kanba S, Teo AR (2018). Hikikomori: experience in Japan and international relevance. World Psychiatry.

[6] Yong RK, Kaneko Y (2016). Hikikomori, a phenomenon of social withdrawal and isolation in young adults marked by an anomic response to coping difficulties: a qualitative study exploring individual experiences from first- and second-person perspectives. Open J Prev Med.

[7] da Silva AC, Cureau FV, de Oliveira CL (2019). Physical activity but not sedentary time is associated with vitamin D status in adolescents: study of cardiovascular risk in adolescents (ERICA). Eur J Clin Nutr.

[8] Vellingiri K, Ethiraj P, S NJ, Shanthappa AH, J KS (2021). Assessment of vitamin D levels and its correlation with osteoporosis and fracture site comminution in osteoporotic hip fractures in tertiary care hospital. Cureus.

[9] Teo AR, Chen JI, Kubo H (2018). Development and validation of the 25-item Hikikomori Questionnaire (HQ-25). Psychiatry Clin Neurosci.

[10] Kato TA, Kanba S, Teo AR (2019). Hikikomori : multidimensional understanding, assessment, and future international perspectives. PCN.

[11] Ratan ZA, Parrish AM, Zaman SB, Alotaibi MS, Hosseinzadeh H (2021). Smartphone addiction and associated health outcomes in adult populations: a systematic review. Int J Environ Res Public Health.

[12] Hassan AA, Elbashir MI, Al-Nafeesah A, AlEed A, Adam I (2026). Association between physical activity and serum 25-hydroxyvitamin D levels among adolescents in Northern Sudan: a school-based cross-sectional study. Nutrients.

[13] Dresp-Langley B, Hutt A (2022). Digital addiction and sleep. Int J Environ Res Public Health.

[14] Schnadower D, Agarwal C, Oberfield SE, Fennoy I, Pusic M (2006). Hypocalcemic seizures and secondary bilateral femoral fractures in an adolescent with primary vitamin D deficiency. Pediatrics.

[15] Coriell MB, Van Hersh AT, Shah S (2021). Prolonged seizure activity followed by severe hyperphosphatemia and hypocalcemia in a pediatric patient. Cureus.

[16] Chandankere V, Mulpur P, Maryada VR, Pendyala SR, Annapareddy GV (2019). Simultaneous bilateral neck of femur fractures in an adolescent secondary to hypocalcaemic seizure. J Pediatr Orthop B.

